# Inhaled remdesivir reduces viral burden in a nonhuman primate model of SARS-CoV-2 infection

**DOI:** 10.1126/scitranslmed.abl8282

**Published:** 2021-12-30

**Authors:** Meghan S. Vermillion, Eisuke Murakami, Bin Ma, Jared Pitts, Adrian Tomkinson, Davin Rautiola, Darius Babusis, Hammad Irshad, Dustin Siegel, Cynthia Kim, Xiaofeng Zhao, Congrong Niu, Jesse Yang, Andrew Gigliotti, Nani Kadrichu, John P. Bilello, Scott Ellis, Roy Bannister, Raju Subramanian, Bill Smith, Richard L. Mackman, William A. Lee, Philip J. Kuehl, Jim Hartke, Tomas Cihlar, Danielle P. Porter

**Affiliations:** ^1^ Lovelace Biomedical; 2425 Ridgecrest Drive, SE, Albuquerque, NM 87108, USA.; ^2^ Gilead Sciences; 333 Lakeside Drive, Foster City, CA 94404, USA.; ^3^ Inspired - Pulmonary Solutions; San Carlos, CA 94070, USA.

## Abstract

Remdesivir (RDV) is a nucleotide analog prodrug with demonstrated clinical benefit in patients with coronavirus disease 2019 (COVID-19). In October 2020, the US FDA approved intravenous (IV) RDV as the first treatment for hospitalized COVID-19 patients. Furthermore, RDV has been approved or authorized for emergency use in more than 50 countries. To make RDV more convenient for non-hospitalized patients earlier in disease, alternative routes of administration are being evaluated. Here, we investigated the pharmacokinetics and efficacy of RDV administered by head dome inhalation in African green monkeys (AGM). Relative to an IV administration of RDV at 10 mg/kg, an approximately 20-fold lower dose administered by inhalation produced comparable concentrations of the pharmacologically active triphosphate in lower respiratory tract tissues. Distribution of the active triphosphate into the upper respiratory tract was also observed following inhaled RDV exposure. Inhalation RDV dosing resulted in lower systemic exposures to RDV and its metabolites as compared with IV RDV dosing. An efficacy study with repeated dosing of inhaled RDV in an AGM model of SARS-CoV-2 infection demonstrated reductions in viral replication in bronchoalveolar lavage fluid and respiratory tract tissues compared with placebo. Efficacy was observed with inhaled RDV administered once daily at a pulmonary deposited dose of 0.35 mg/kg beginning approximately 8 hours post-infection. Moreover, the efficacy of inhaled RDV was similar to that of IV RDV administered once at 10 mg/kg followed by 5 mg/kg daily in the same study. Together, these findings support further clinical development of inhalation RDV.

## Teaser

Inhaled remdesivir (RDV) reduces viral burden in nonhuman primates infected with SARS-CoV-2.

## INTRODUCTION

Coronaviruses (CoVs) are positive-sense, single-stranded, enveloped RNA viruses, many of which commonly infect humans and cause mild symptoms. However, over the past two decades, emerging pathogenic CoVs that can cause life-threatening disease in humans and animals have been identified, namely severe acute respiratory syndrome coronavirus (SARS-CoV) (*1*, *2*), Middle Eastern respiratory syndrome coronavirus (MERS-CoV) (*3*–*5*), and SARS-CoV-2, the causative agent of coronavirus disease 2019 (COVID-19) (*6*). The global pandemic caused by SARS-CoV-2 has resulted in severe respiratory illness throughout the world. Severe cases progress to pneumonia and multi-organ failure, which can lead to death (*7*). Though advances in vaccine development and non-pharmaceutical interventions have dramatically reduced the spread of SARS-CoV-2, therapeutic options to prevent disease progression are limited, and effective treatments that can be administered outside of healthcare settings are currently not readily available. Continued development and evaluation of effective COVID-19 treatments that can be administered in non-hospitalized settings is, therefore, of urgent and critical importance.

Remdesivir (RDV; GS-5734; Veklury) is a single diastereomer monophosphoramidate prodrug of an adenosine analog that was first discovered as a potent inhibitor of respiratory syncytial virus (*8*). It is intracellularly metabolized by host enzymes – including cathepsin A (CatA), carboxylesterase 1 (CES1), and histidine triad nucleotide-binding protein 1 (HINT1) – to its pharmacologically active triphosphate form (RDV-TP; GS-443902), which in turn acts as a potent and selective inhibitor of multiple viral RNA polymerases (*9*, *10*).

In October 2020, the US FDA approved RDV for the treatment of COVID-19 in hospitalized adult and pediatric patients (12 years of age and older and weighing at least 40 kg) (*11*). Worldwide, RDV has been approved or authorized for emergency use in more than 50 countries. In current clinical use, RDV is administered intravenously (IV) to hospitalized patients, which limits its use early in the disease course. To more readily enable administration to non-hospitalized patients earlier in the course of infection, alternative routes of administration are being evaluated to enable broader distribution and home use. Because respiratory epithelial cells expressing the angiotensin converting enzyme 2 (ACE2) receptor represent the initial targets of SARS-CoV-2 infection (*12*), delivering RDV directly to the primary site of infection with a nebulized, inhaled solution may enable more targeted and accessible administration in non-hospitalized patients. In addition, local administration directly to the respiratory tract might lower systemic exposure to the drug, further reducing systemic exposure of RDV. Here, we report on the pharmacokinetics (PK), biodistribution, safety, and efficacy of an inhaled solution of RDV assessed in the preclinical African green monkey (AGM) model of SARS-CoV-2 infection.

## RESULTS

### Single-dose PK and biodistribution of RDV administered by head dome inhalation or IV infusion in non-infected AGM

**Fig. 1.**
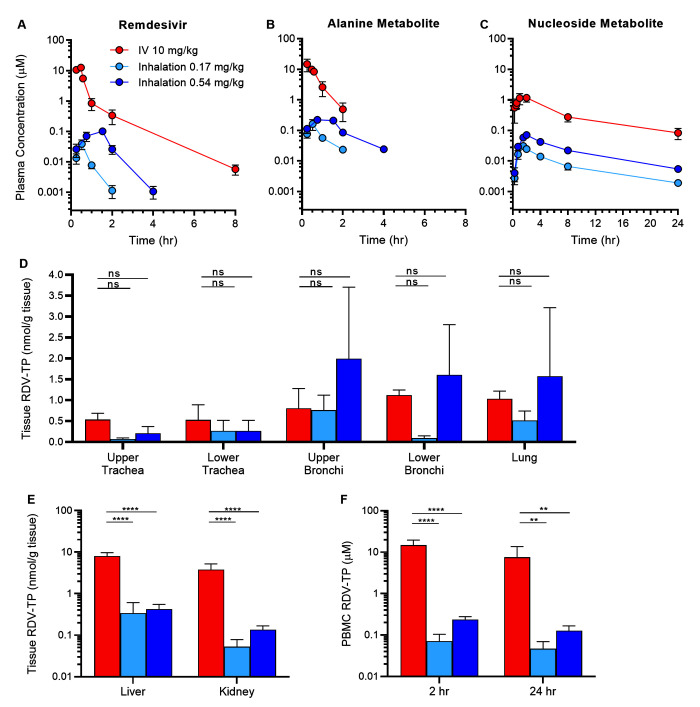
Inhaled remdesivir (RDV) results in similar RDV-TP concentrations in the respiratory tract despite lower systemic and non-respiratory exposures. Plasma concentrations of (**A**) RDV and its (**B**) alanine metabolite and (**C**) nucleoside metabolite were determined after a single dose of RDV administered by intravenous (IV, 10 mg/kg) or inhalation (0.17 or 0.54 mg/kg deposited dose) routes to AGM (n=3 to 4 per treatment group). Tissue concentrations of the active triphosphate metabolite RDV-TP were measured in (**D**) respiratory tissues, (**E**) liver and kidney, (**F**) and PBMCs approximately 24 hours post-exposure. Values are presented as mean ± SD. ns: not significant, **p <0.01, ****p <0.0001; data were analyzed using a repeated measures two-way ANOVA with Bonferroni post-hoc correction.

The single-dose PK and biodistribution of RDV and key metabolites were determined after administration of RDV by head dome inhalation (30 or 90 min) or IV infusion in healthy AGM (Table 1, Fig. 1A to C). RDV (5 mg/mL solution, identical to the IV formulation) was nebulized using a compressed air nebulizer to produce an aerosol with an average concentration of 48 μg/L and an average particle size of 2.7 μm. Standard methods (such as aerosol concentration analysis and an estimated 25% deposition fraction of the presented aerosol to the lung (*13*)) were used to characterize the head dome delivery of this aerosol to alert, free-breathing AGM (fig. S1). Exposure of AGM to aerosolized RDV for 30 or 90 min resulted in estimated pulmonary deposited doses of 0.17 mg/kg and 0.54 mg/kg, respectively. Intravenous RDV was delivered as a constant rate infusion over 30 min for a total dose of 10 mg/kg. This dose in AGM approximates a human equivalent loading dose of RDV (200 mg IV) (*14*) and was selected based on previous evaluation of this loading dose administered IV in a rhesus macaque SARS-CoV-2 infection model (*15*). Systemic or intracellular metabolites of RDV include an alanine metabolite (GS-704277) and a nucleoside metabolite (GS-441524), in addition to intracellular, mono- and di-phosphate metabolites (fig. S2).

**Table 1.** Systemic pharmacokinetic parameters of remdesivir (RDV) and key metabolites were measured after intravenous (IV) and inhalation routes of administration. Plasma concentrations of RDV and its alanine metabolite and nucleoside metabolite were measured after a single dose of RDV administered to AGM by IV (10 mg/kg; n=3) or inhalation (5 mg/mL for 30 or 90 min resulting in deposited doses of 0.17 and 0.54 mg/kg, respectively, n=4 per dose) routes. C_max_ and AUC_last_ values presented are the mean ± SD for each analyte and treatment group. Data for each analyte were analyzed using a one-way ANOVA with Dunnett post-hoc correction, and presented *p* values represent comparison of each inhalation dose group with the IV dose group.

**Analyte**	**RDV Route, Dose**	**Plasma PK Parameter**			
		**C_max_ (μM)**	***p* Value**	**AUC_last_ (μM•hr)**	***p* Value**
Remdesivir	IV, 10 mg/kg	12.6 ± 1.0	N/A	7.26 ± 1.29	N/A
	Inhalation, 0.17 mg/kg	0.04 ± 0.01	<0.0001	0.02 ± 0.01	<0.0001
	Inhalation, 0.54 mg/kg	0.10 ± 0.02	<0.0001	0.15 ± 0.05	<0.0001
Alanine metabolite	IV, 10 mg/kg	15.9 ± 4.9	N/A	9.47 ± 1.07	N/A
	Inhalation, 0.17 mg/kg	0.16 ± 0.06	<0.0001	0.13 ± 0.03	<0.0001
	Inhalation, 0.54 mg/kg	0.23 ± 0.03	<0.0001	0.42 ± 0.05	<0.0001
Nucleoside metabolite	IV, 10 mg/kg	1.23 ± 0.36	N/A	9.06 ± 2.60	N/A
	Inhalation, 0.17 mg/kg	0.03 ± 0.01	<0.0001	0.19 ± 0.03	<0.0001
	Inhalation, 0.54 mg/kg	0.07 ± 0.01	<0.0001	0.54 ± 0.08	<0.0001

Inhalation of RDV at both 0.17 mg/kg and 0.54 mg/kg deposited doses resulted in dose-dependent systemic exposures – both maximum concentration (C_max_) and area under the plasma time-correction curve (AUC_last_) – to RDV and its alanine and nucleoside metabolites that were lower than systemic exposures after 10 mg/kg IV administration (Table 1). Following intravenous infusion of RDV at 10 mg/kg, a biphasic profile was observed in plasma with rapid distribution and elimination phases and a mean terminal half-life of 1.0 hour (Fig. 1A). The alanine metabolite also appeared and declined rapidly from plasma (Fig. 1B). The nucleoside metabolite appeared in plasma more slowly and at lower concentrations but persisted over the 24-hour sampling period with a mean terminal half-life of 6.2 hours (Fig. 1C).

Following inhaled deposited doses of RDV at 0.17 or 0.54 mg/kg in AGM, RDV appeared in plasma during aerosol exposure and cleared rapidly with mean half-lives of 0.27 and 0.34 hours, respectively (Fig. 1A). The plasma exposure to RDV (C_max_ and AUC_last_) increased with the inhaled deposited dose (Table 1). During aerosol RDV exposure, plasma concentrations of the alanine metabolite exceeded plasma concentrations of RDV, and then declined rapidly with mean half-lives of 0.53 and 0.89 hours, for the 0.17 and 0.54 mg/kg inhaled deposited doses, respectively (Fig. 1B). The nucleoside metabolite appeared more gradually in plasma and persisted over the 24-hour sampling period with mean estimated terminal elimination half-lives of 7.58 and 7.10 hours, respectively (Fig. 1C).

Following exposure to the RDV prodrug, intracellular metabolism results in production of RDV-MP, followed by production of the active metabolite, RDV-TP. Approximately 24 hours following inhalation RDV delivery, pulmonary deposited doses of 0.17 or 0.54 mg/kg resulted in dose-dependent respiratory tissue exposures to the pharmacologically active RDV-TP. RDV-TP concentrations in the conducting airways and lung tissue following pulmonary delivery of 0.54 mg/kg RDV were comparable to those observed after IV dosing of 10 mg/kg RDV (Fig. 1D and table S1). Following inhalation administration, RDV-TP was also quantifiable in both nasal and nasopharyngeal mucosa with relative concentrations that increased with dose (fig. S3).

In non-respiratory tissues (Fig. 1E) and peripheral blood mononuclear cells (PBMC, Fig. 1F), inhaled delivery of either 0.17 or 0.54 mg/kg RDV resulted in lower concentrations of RDV-TP as compared with IV delivery at 10 mg/kg RDV (table S1). Following inhaled administration at 0.54 mg/kg, RDV-TP concentrations in the liver, kidney, and 24 hour PBMC samples were approximately 19-, 28-, and 59-fold lower than the corresponding concentrations following IV administration at 10 mg/kg. Inhaled RDV was well-tolerated by all animals, and there were no gross or microscopic findings within the respiratory tract that could be attributed to RDV dosing in this study.

### Characterization of the SARS-CoV-2 AGM model

**Fig. 2.**
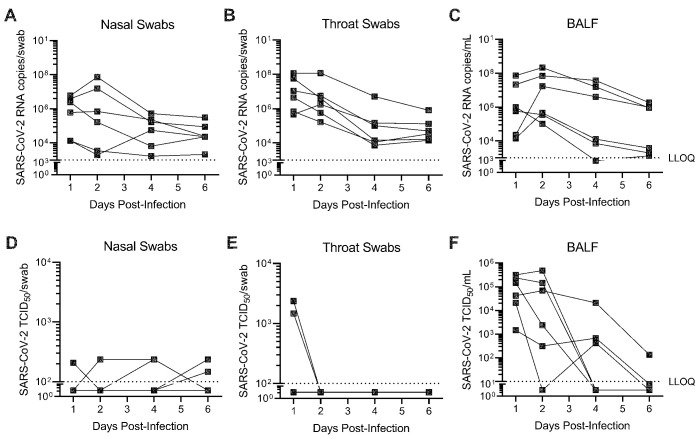
SARS-CoV-2 replicates in both the upper and lower respiratory tract of AGM. AGM (n=6) were infected with SARS-CoV-2 by intranasal and intratracheal instillation. SARS-CoV-2 (**A to C**) RNA and (**D to F**) infectious viral titers were quantified from nasal (**A, D**) and throat (**B**, **E**) swabs, as well as bronchoalveolar lavage fluid (BALF) (**C, F**) collected through 6 days post-infection. SARS-CoV-2 RNA copies were quantified by RT-qPCR. Infectious SARS-CoV-2 titers were determined by a TCID_50_ assay. Each connected line represents an individual animal. LLOQ indicates lower limit of quantification.

**Fig. 3.**
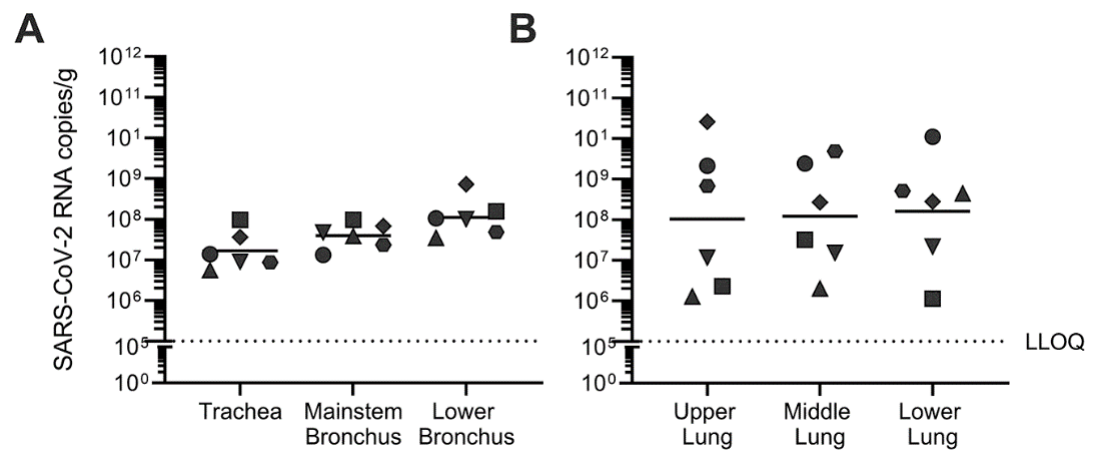
SARS-CoV-2 infection is evenly distributed across the conducting airways and lung lobes collected six days post-infection. AGM (n=6) were infected with SARS-CoV-2 by intranasal and intratracheal instillation. SARS-CoV-2 RNA was quantified in (**A**) conducting airways and (**B**) lung tissue at 6 days post-infection by RT-qPCR. Individual animals are represented by unique symbols. Horizontal bars represent the mean value for each tissue. LLOQ indicates lower limit of quantification.

SARS-CoV-2 replication and clinical disease was characterized in AGM inoculated with 3×10^6^ median tissue culture infectious dose (TCID_50_) SARS-CoV-2 by combined intranasal and intratracheal (IN/IT) instillation. SARS-CoV-2 RNA was detectable in serially collected nasal swabs, throat swabs, and bronchoalveolar lavage fluid (BALF) out to 6 days post-inoculation (dpi), with peak viral load in most animals occurring 1 to 2 dpi (Fig. 2A to C). The majority of nasal and throat swabs were negative for infectious SARS-CoV-2, as measured by median TCID_50_ assay (Fig. 2D and E). Infectious virus in BALF was detectable in the majority of animals at both 1 and 2 dpi, with infectious titers quantified between approximately 10^3^ to 10^6^ TCID_50_/mL (Fig. 2F). Respiratory tract tissues collected 6 dpi had quantifiable SARS-CoV-2 RNA in both conducting airways (Fig. 3A) and lung tissue (Fig. 3B), with similar distribution of virus between upper, middle and lower lung lobes within each animal.

Consistent with previous reports (*16*), clinical disease following SARS-CoV-2 infection in AGM was overall mild in the acute phase. Throughout the course of the 6-day study, body weights and body temperatures remained unchanged from baseline and within normal ranges (fig. S4A and B). Thoracic radiographs captured at 2, 4, and 6 dpi showed no overt lung pathology (fig. S4C). Microscopic lesions within the respiratory tract were also mild and were characterized by mild focal or multifocal mixed cell inflammation with fibrin and epithelial hypertrophy/hyperplasia (fig. S4D).

### Efficacy and PK of inhaled RDV in the SARS-CoV-2 AGM model

The efficacy of inhaled and IV RDV in the SARS-CoV-2 AGM model was evaluated in animals infected with 3×10^6^ TCID_50_ SARS-CoV-2 by IN/IT instillation. Starting at approximately 8 hours post-inoculation, animals were treated with RDV or vehicle by either IV (10 mg/kg loading dose followed by 5 mg/kg daily maintenance doses for 6 days [10/5 mg/kg]) or daily inhalation (0.35 mg/kg deposited dose) dosing for 6 days. The target inhalation dose corresponded with a 60-min head dome exposure, and was projected to yield approximately equivalent RDV-TP concentrations in the lung as a 10 mg/kg IV dose based on the PK study results.

Treatment with either inhaled or IV RDV was well tolerated by all animals. Mild body weight decreases in the first 2 dpi were observed in all groups and were likely attributed to daily anesthetized procedures in the beginning of the study (fig. S5). Clinical observations were limited to a single observation of cough in a vehicle animal that was not associated with any other respiratory signs.

Intravenous dosing of RDV resulted in average peak plasma concentrations of 3.7 μM, whereas inhalation dosing of RDV resulted in average peak plasma concentrations of 0.1 μM (fig. S6). In both treatment groups, trough plasma RDV concentrations were below the assay limit of quantification. Peak plasma concentrations of the nucleoside metabolite averaged 0.28 and 0.05 μM for the IV and inhalation treatment groups, respectively. Peak plasma concentrations of the alanine metabolite averaged 1.4 and 0.2 μM for the IV and inhalation treatment groups, respectively, with trough concentrations for both groups below the limit of quantification. The peak plasma concentrations of RDV and its two metabolites in SARS-CoV-2-infected AGM were similar to those observed in uninfected AGM (Fig. 1A to C). RDV and metabolites in BALF samples (collected approximately 18 to 20 hours post-dose) were below the limit of quantification in both treatment groups.

**Fig. 4.**
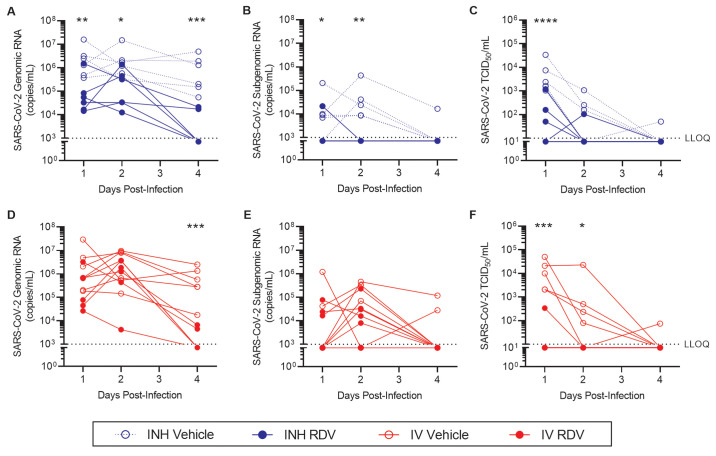
Treatment with inhaled RDV following SARS-CoV-2 infection reduces viral load in bronchoalveolar lavage fluid. AGM were inoculated with SARS-CoV-2 and treated with (**A to C**) inhaled or (**D to F**) IV RDV (closed symbols) or vehicle (open symbols) beginning 8 hours post-infection (n=6 per group). Bronchoalveolar lavage fluid was collected 1, 2 and 4 days post-inoculation. SARS-CoV-2 genomic (**A, D**) and subgenomic (**B, E**) RNA copies were quantified by RT-qPCR. Infectious SARS-CoV-2 titers (**C, F**) were determined by a TCID_50_ assay. Samples that were below the lower limit of quantification (LLOQ, dotted lines) for the assay were assigned the LLOQ for analyses. *p <0.05, **p <0.01, ***p <0.001, ****p <0.0001; data were analyzed by a repeated measures two-way ANOVA with Bonferroni post-hoc correction.

We assessed the impact of inhaled RDV (Fig. 4A to C) and IV administered RDV (Fig. 4D to F) on reduction in viral genomic RNA, subgenomic RNA, and infectious virus. Compared with respective vehicle-treated controls, inhaled RDV treatment resulted in reductions in SARS-CoV-2 genomic RNA in BALF collected 1, 2 and 4 dpi (Fig. 4A), and reductions in SARS-CoV-2 subgenomic (sg) RNA collected 1 and 2 dpi (Fig. 4B). In BALF collected 1 dpi, inhaled RDV reduced SARS-CoV-2 RNA copies by an average 1.5 logs, whereas IV RDV reduced SARS-CoV-2 RNA copies by an average 0.8 logs, as compared with the respective vehicle controls (Table 2). Infectious SARS-CoV-2 titers, as measured by TCID_50_ assay in BALF collected 1 dpi, were reduced by treatment with both inhaled and IV RDV (Fig. 4C, F), with an average magnitude of 1.9 and 2.2 logs, respectively (Table 2).

**Table 2.** SARS-CoV-2 RNA and infectious viral titers were reduced in bronchoalveolar lavage fluid after IV or inhalation RDV treatment. SARS-CoV-2 genomic (g) RNA copies, subgenomic (sg) RNA copies, and infectious viral titers were measured in bronchoalveolar lavage fluid (BALF) collected 1, 2, and 4 days post-infection (dpi) from AGM dosed with either RDV or vehicle by either inhalation (INH) or intravenous (IV) administration beginning 8 hours post-infection with SARS-CoV-2 (n=6 per group). Group means, mean differences, and *p* values based on repeated measures two-way ANOVA with Bonferroni post-hoc correction are presented.

**dpi**	**Group**	**Mean log (copies/mL)** **g RNA**	**Mean Difference**	***p* Value**	**Mean log (copies/mL)** **sg RNA**	**Mean Difference**	***p* Value**	**Mean log (TCID_50_/mL)**	**Mean Difference**	***p* Value**
1	INH RDV	4.790	-1.452	0.0032	3.222	-0.851	0.0394	1.662	-1.872	<0.0001
	INH Vehicle	6.242			4.073			3.533		
	IV RDV	5.353	-0.795	0.3035	3.748	-0.036	>0.9999	1.255	-2.188	0.0001
	IV Vehicle	6.148			3.784			3.443		
2	INH RDV	5.075	-1.102	0.0299	3.000	-1.251	0.0016	1.170	-0.602	0.2619
	INH Vehicle	6.177			4.251			1.772		
	IV RDV	5.793	-0.462	>0.9999	4.246	0.075	>0.9999	1.000	-1.222	0.0383
	IV Vehicle	6.255			4.171			2.222		
4	INH RDV	3.428	-2.290	<0.0001	3.000	-0.203	>0.9999	1.000	-0.117	>0.9999
	INH Vehicle	5.718			3.203			1.117		
	IV RDV	3.245	-2.328	<0.0001	3.000	-0.589	0.9040	1.000	-0.145	>0.9999
	IV Vehicle	5.573			3.587			1.145		

At 4 dpi, both inhaled and IV RDV treatment reduced SARS-CoV-2 RNA in BALF (Fig. 4A, D), with an average reduction of 2.3 logs compared with respective vehicle controls, assuming negative samples expressed SARS-CoV-2 RNA at the assay limit of detection (Table 2). At this timepoint, only 2 of 6 animals in each RDV treatment group had detectable SARS-CoV-2 viral RNA in BALF, regardless of administration route, whereas BALF from all 6 animals in each vehicle treatment group was PCR positive for SARS-CoV-2. The majority of BALF samples collected 4 dpi were below the assay limit of quantification for SARS-CoV-2 sgRNA (Fig. 4B, E) and infectious SARS-CoV-2 (Fig. 4C, F).

We measured changes in nasal (fig. S7A to D) and throat (fig. S7E to H) genomic and subgenomic RNA after inhaled and IV RDV treatment. SARS-CoV-2 genomic RNA detected in nasal swabs was variable. In most animals, peak viral RNA was observed 1 to 2 dpi, with no differences observed between RDV and vehicle treated animals by either the inhalation or IV route (fig. S7A, C). Subgenomic RNA was undetectable in all but one nasal swab (fig. S7B, D). In throat swabs, treatment with inhaled RDV resulted in a reduction in genomic SARS-CoV-2 measured 2 dpi (fig. S7E), but subgenomic RNA was not quantifiable at this timepoint (fig. S7F). No treatment effect was observed with IV RDV treatment in throat swabs (fig. S7G, H).

**Fig. 5.**
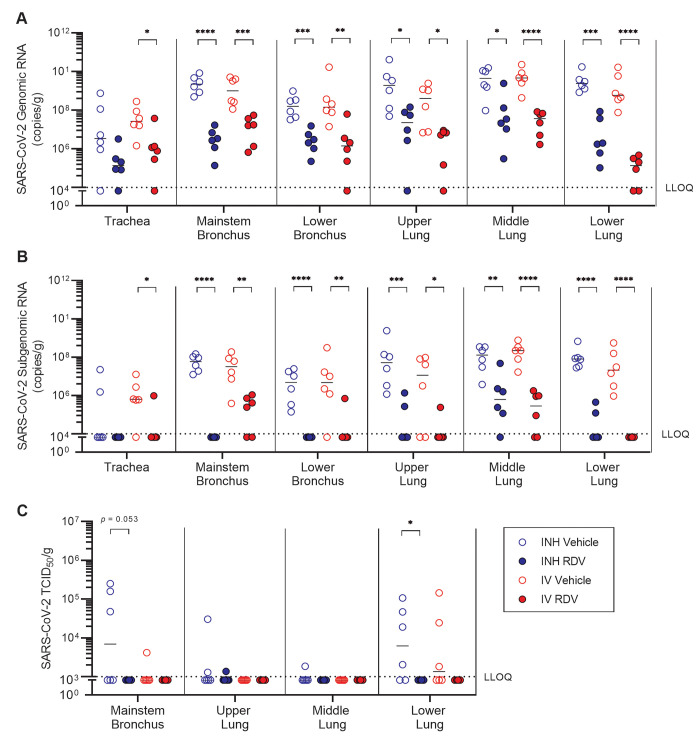
Treatment with inhaled RDV following SARS-CoV-2 infection reduces viral load in respiratory tract tissues. AGM were inoculated with SARS-CoV-2 and treated with inhaled (blue symbols) or IV (red symbols) RDV (closed symbols) or vehicle (open symbols) beginning at 8 hours post-infection (n=6 each). Respiratory tissues were collected 6 days post-inoculation. SARS-CoV-2 (**A**) genomic and (**B**) subgenomic RNA copies were quantified by RT-qPCR. (**C**) Infectious SARS-CoV-2 titers were determined by a TCID_50_ assay. Samples that were below the lower limit of quantification (LLOQ, dotted lines) for the assay were assigned a value equal to the LLOQ for analyses. Horizontal bars indicate median values. *p <0.05, **p <0.01, ***p <0.001, ****p <0.0001; data were analyzed by a repeated measures two-way ANOVA with Bonferroni post-hoc correction.

At 6 dpi, genomic SARS-CoV-2 RNA was quantifiable in almost all respiratory tissues analyzed (Fig. 5A), and subgenomic RNA was detectable in almost all samples from vehicle treatment groups (Fig. 5B). With the exception of the trachea, where viral RNA copy numbers were the lowest, both IV and inhaled remdesivir treatment resulted in reductions in viral genomic and subgenomic RNA (Fig. 5A and B). The effect of treatment on viral load was similar between IV and inhaled treatment routes (Fig. 5A and B). In each of these tissues, the average treatment-associated reduction in genomic SARS-CoV-2 ranged from 1.8 to 2.9 logs compared with respective vehicle controls (Table 3). The largest overall effect of treatment was observed in the lower lung, where inhaled and IV remdesivir treatment resulted in an average 4.1 and 3.0 log reduction in genomic SARS-CoV-2 RNA, respectively (Fig. 5A, Table 3), and an average 3.3 and 3.5 log reduction in subgenomic SARS-CoV-2 RNA, respectively (Fig. 5B, Table 3). This was accompanied by an effect of both inhaled and IV RDV treatment in reducing infectious virus load in the lower lung (Fig. 5C). Consistent with pathology observed in the AGM model development study (fig. S4C), pulmonary inflammation in this infection model was generally mild, characterized by mixed cell inflammation with alveolar epithelial hypertrophy/hyperplasia, which was present in all vehicle-treated animals (table S2). Overall, treatment with either IV or inhaled RDV decreased the incidence and severity of pulmonary inflammation compared with respective vehicle controls (table S2).

**Table 3.** SARS-CoV-2 RNA was reduced in respiratory tract tissues after IV or inhalation RDV treatment. SARS-CoV-2 genomic (g) RNA and subgenomic (sg) RNA copies were measured in trachea, mainstem bronchus, lower bronchus, upper lung, middle lung, and lower lung collected 6 days post-infection (dpi) from AGM dosed with either RDV or vehicle by either inhalation (INH) or intravenous (IV) administration beginning 8 hours post-infection with SARS-CoV-2 (n=6 per group). Group means, mean differences, and *p* values based on repeated measures two-way ANOVA with Bonferroni post-hoc correction are presented.

**Tissue**	**Group**	**Mean log(copies/mL)** **g RNA**	**Mean Difference**	***p* Value**	**mean log(copies/mL)** **sg RNA**	**Mean Difference**	***p* Value**
Trachea	INH RDV	5.197	-1.467	0.0870	4.000	0.9232	0.1568
	INH Vehicle	6.663			4.923		
	IV RDV	5.855	-1.578	0.0196	4.331	-1.486	0.0198
	IV Vehicle	7.433			5.817		
Mainstem bronchus	INH RDV	6.373	-2.943	<0.0001	4.000	-3.683	<0.0001
	INH Vehicle	9.317			7.683		
	IV RDV	6.950	-2.018	0.0009	5.150	-2.128	0.0030
	IV Vehicle	8.968			7.278		
Lower bronchus	INH RDV	6.348	-1.832	0.0004	4.000	-2.447	<0.0001
	INH Vehicle	8.180			6.447		
	IV RDV	5.835	-2.523	0.0089	4.308	-2.217	0.0088
	IV Vehicle	8.358			6.525		
Upper lung	INH RDV	6.865	-2.252	0.0153	4.594	-2.995	0.0007
	INH Vehicle	9.117			7.589		
	IV RDV	6.090	-2.143	0.0105	4.231	-2.102	0.0256
	IV Vehicle	8.233			6.332		
Middle lung	INH RDV	7.483	-1.970	0.0102	5.780	-2.111	0.0056
	INH Vehicle	9.453			7.891		
	IV RDV	7.302	-2.310	<0.0001	5.191	-3.014	<0.0001
	IV Vehicle	9.612			8.205		
Lower lung	INH RDV	6.475	-2.962	0.0001	4.457	-3.474	<0.0001
	INH Vehicle	9.437			7.932		
	IV RDV	4.905	-4.088	<0.0001	4.000	-3.340	<0.0001
	IV Vehicle	8.993			7.340		

## DISCUSSION

Remdesivir is currently administered by IV, which limits its use to individuals with advanced disease who have been admitted to hospitals or intensive care units for clinical COVID-19, and precludes earlier treatment of patients following SARS-CoV-2 infection, prior to the onset of severe disease symptoms. The NIH-sponsored ACTT-1 study showed that patients treated with IV RDV experienced a shortened time to clinical improvement (*17*), but the clinical benefit excluded those requiring mechanical ventilation, suggesting that the efficacy of RDV is greater for those with mild to moderate disease. More recent data from a randomized, placebo-controlled phase 3 clinical trial in non-hospitalized, high-risk individuals with COVID-19 demonstrated a significant reduction in hospitalizations with a 3-day course of IV remdesivir (*18*). These data suggest that early treatment prior to hospitalization maximizes therapeutic benefit by limiting viral dissemination and subsequent disease progression.

To more readily enable administration to non-hospitalized patients earlier in disease, an inhaled formulation of RDV that was identical to the IV formulation was characterized and tested for in vivo efficacy. Local inhalation delivery has the potential for targeted dosing that achieves similar concentrations of active metabolites in respiratory tract tissues when delivered at lower doses. In this study, the PK, biodistribution and safety of an inhaled solution of RDV was assessed in AGM, and efficacy was evaluated following high-titer SARS-CoV-2 challenge.

Compared with an IV dose of 10 mg/kg, an approximately 20-fold lower inhalation dose resulted in, on average, 53% higher lung concentrations of the active triphosphate RDV-TP with approximately 50-fold lower systemic exposures to RDV. Inhalation administration of RDV to AGM also resulted in comparable concentrations of RDV-TP in the conducting airways, including the upper and lower trachea and upper and lower bronchus. Compared with IV administration, inhaled RDV delivery resulted in lower exposures to RDV metabolites in non-respiratory tissues (liver, kidney and PBMC). Plasma exposure of RDV in AGM following a single IV dose at 10 mg/kg (AUC, 7.26 μM•hr) was similar to that observed in healthy human volunteers at 200 mg (AUC, 4.75 μM•hr), suggesting that 10 mg/kg RDV IV in AGM delivers equivalent systemic exposures to approximately 200 mg IV dose in humans, which is the clinical loading dose (*14*). Thus, the RDV-TP loading at 200 mg IV in humans is equivalent to 0.35 mg/kg inhalation in AGM, assuming the lung loading efficiency is similar between humans and monkeys.

An AGM model of SARS-CoV-2 infection was established that supports viral replication without overt signs of clinical disease and pathology, similar to the majority of SARS-CoV-2 cases in humans. The viral replication kinetics and viral loads in upper and lower respiratory samples from this model were similar to those reported from other studies that describe SARS-CoV-2 infection in AGM by multiple delivery routes (*16*, *19*). Daily treatment of AGM with either 0.35 mg/kg RDV (deposited dose) by head dome inhalation or with 10/5 mg/kg RDV by IV infusion beginning approximately 8 hours after virus inoculation resulted in a reduction in SARS-CoV-2 load across the respiratory tract. Treatment-associated reductions in SARS-CoV-2 genomic RNA and infectious virus were observed as early as 1 dpi (approximately 16 hours following initiation of treatment). In BALF collected 1 dpi, IV and inhaled RDV treatment reduced infectious SARS-CoV-2 titers by 2.2 and 1.9 logs, respectively, compared with the corresponding vehicle controls. In respiratory tract tissues, treatment-associated reductions in genomic SARS-CoV-2 RNA were observed in both conducting airways (mainstem and lower bronchus) and in upper, middle, and lower lung tissues. The magnitude of the treatment effect in the conducting airways ranged from 1.8 to 2.9 logs. In the lower lung tissues, treatment with either IV or inhaled RDV resulted in an average reduction of genomic SARS-CoV-2 RNA by 4.1 or 3.0 logs, respectively. Comparing the mainstem bronchus with the lower lung, inhaled RDV treatment demonstrated nearly equivalent efficacy in both tissues, whereas the effect of IV RDV treatment was more than twice as large in the lower lung than in the mainstem bronchus.

Pulmonary inflammation, albeit mild overall in this model, was less frequently observed in animals treated with RDV compared with vehicle controls. An effect of RDV treatment on viral load in samples collected from the upper respiratory tract (nasal swabs, throat swabs, and trachea), however, was limited to efficacy observed for inhaled RDV on genomic viral RNA in throat swabs collected 2 dpi. These data suggest that RDV administered by inhalation may limit dissemination of virus to the lower airways or attenuate viral replication in the lower airways, which may be associated with reduced pulmonary inflammation despite minimal effect on viral load in the upper respiratory tract. Overall, the results suggest that the antiviral effects of a lower dose (0.35 mg/kg) administered by inhalation are similar to those of 10/5 mg/kg IV RDV, consistent with comparable concentrations of active triphosphate in the lung and bronchus tissues.

Our study has several limitations. The efficacy of inhaled remdesivir presented here is based on initiation of treatment approximately 8 hours following SARS-CoV-2 inoculation. Though it should be acknowledged that therapeutic intervention within this short window may not be practical in a clinical setting, this study design was based on characterization of SARS-CoV-2 infection in nonhuman primate models (*16*, *20*–*22*), in which peak viral replication occurs between 1 to 3 days post-infection, and virus is cleared from most respiratory samples by 10 days post-infection. The rapid SARS-CoV-2 replication in nonhuman primates, therefore, necessitates early therapeutic intervention in order to evaluate efficacy of an antiviral such as remdesivir, which targets replicating virus. The studies described herein demonstrate efficacy of inhaled remdesivir against ancestral lineage SARS-CoV-2 (USA-WA1/2020 isolate). Since the conduct of these experiments, several SARS-CoV-2 variants, including the Delta variant, have emerged as the predominant circulating strains. Remdesivir retains in vitro antiviral activity against the Alpha, Beta, Gamma, Epsilon, and Delta variants with potency comparable to the ancestral lineage WA1 reference isolate used in the current study (*23*). Although not specifically evaluated in this study, it is reasonable to expect that similar results would be obtained in studies using the Delta variant.

Collectively, these preclinical data in AGM demonstrate that delivery of RDV by inhalation achieves effective antiviral activity in the respiratory tract following SARS-CoV-2 infection. Moreover, inhaled RDV resulted in lower systemic exposures than IV administration, and was well tolerated as a repeat-dose regimen during SARS-CoV-2 infection. Inhaled RDV, therefore, is a safe and effective therapeutic approach for controlling viral load during early SARS-CoV-2 infection. Further, refinement of an inhaled RDV formulation that can be distributed for home use would expand access of an effective antiviral to non-hospitalized patients and enable earlier therapeutic intervention. By overcoming these barriers associated with the current standard of care, inhaled RDV represents a promising treatment strategy for reducing the COVID-19 public health burden.

## MATERIALS AND METHODS

### Study design

The single-dose PK of RDV and key metabolites after a single dose administration of RDV by IV infusion or inhalation was determined in AGM. RDV was administered either by constant-rate IV infusion of 10 mg/kg RDV over 30 min (3 males, 5.6-7.6 kg), or by head dome inhalation of a solution of 5 mg/mL RDV for 30 or 90 min (3 males and 1 female per dose group; 4.8 to 6.2 kg males, 3.6 and 3.9 kg females) (table S3). Blood samples for plasma and PBMCs were collected from a femoral vein or artery and were taken from each monkey over a 24-hour period. Plasma samples from the IV infusion group were obtained at pre-dose and at 0.25, 0.48 (prior to the end of the 30-min infusion), 0.58, 1, 2, 8, and 24 hours post-dose. Plasma samples from the 30-min inhalation group were obtained at pre-dose and at 0.25, 0.52 (immediately after end of the 30-min exposure), 1, 2, 4, 8, and 24 hours post-dose. Plasma samples from the 90-min inhalation group were obtained at pre-dose and at 0.25, 0.75, 1.52 (immediately after end of the 90-min exposure), 2, 4, 8, and 24 hours post-dose. PBMC samples were obtained from all groups at 2 and 24 hours. At approximately 24 hours post-dose, tissues were collected as a terminal procedure. Concentrations of RDV and its two major metabolites, GS-441524 (an adenosine nucleoside analog) and GS-704277 (an intermediate metabolite) were determined in plasma by liquid chromatography with tandem mass spectrometry (LC-MS/MS). Concentrations of GS-441524 and its phosphorylated metabolites were also determined in PBMCs and tissues by LC-MS/MS.

The SARS-CoV-2 AGM model was characterized in 6 animals (3 males and 3 females, 3.6-6.6 kg). On Day 0, animals were infected with SARS-CoV-2 (3×10^6^ TCID_50_/mL) by intranasal (IN, 1 mL; 0.5 mL each nostril) and intratracheal (IT, 2 mL) instillations under anesthesia for a total dose of approximately 3×10^6^ TCID_50_ of virus. Animals were monitored daily for clinical evidence of disease. BALF and nasal and throat swabs were collected 1, 2, 4, and 6 dpi for quantification of infectious viral titers and viral RNA. Thoracic radiographs were taken 2, 4 and 6 dpi under anesthesia using a Dragon X Portable X-Ray unit (TXR). Body weight and temperature were recorded 1, 2, 4, and 6 dpi. Animals were euthanized 6 dpi for collection of respiratory tissue to quantify tissue viral burden and for histopathology.

The efficacy of inhaled and IV RDM in the SARS-CoV-2 AGM model was evaluated in 24 animals (8 males and 16 females, 3.5-6.6 kg; 3 cohorts of 8 animals staggered by 1 day each) (table S4). On Day 0, animals were infected with SARS-CoV-2 by IN/IT instillation. IN (1 mL; 0.5 mL each nostril) and IT (2 mL) instillations were conducted under anesthesia for a total dose of approximately 3×10^6^ TCID_50_ of virus. Starting at approximately 8 hours post-inoculation, animals were treated with RDV or vehicle by either IV or head dome inhalation dosing (n=6 animals/treatment). After the initial RDV dose at 8 hours post-inoculation, subsequent RDV dosing occurred at approximately 28 hours post-inoculation and continued once daily thereafter (every ~24 hours) for 5 additional days (a total of 6 doses per animal). Animals administered IV RDV received an initial dose of 10 mg/kg, followed by daily doses at 5 mg/kg. Animals administered RDV by head dome received an average daily deposited dose of 0.35 mg/kg. Animals were monitored daily for clinical evidence of disease. BALF and nasal and throat swabs were collected 1, 2, 4, and 6 (swabs only) dpi for quantification of infectious viral titers and viral RNA. Body weight and temperature was recorded 1, 2, 4, and 6 dpi. On dosing days, two samples were collected from each animal, one immediately prior to dosing, and one at the end of dosing, to determine trough and peak plasma RDV concentrations, respectively. Animals were euthanized 6 dpi for collection of respiratory tissue to quantify tissue viral burden and for histopathology.

For the PK and efficacy studies, animals were randomized into treatment groups based on sex and pre-study body weight. For the efficacy study, animals were further randomized into three study cohorts such that each treatment group was equally represented within each cohort. To eliminate potential for cross contamination between vehicle and treatment groups, the order of treatments and sample collections was not randomized. Study personnel were not blinded to treatment groups.

### Reagents and formulations

Remdesivir was supplied as a lyophilized powder containing 105 mg of RDV (3.23% w/w) and 3,146 mg of sulfobutylether β-cyclodextrin (SBECD) sodium salt (betadex sulfobutylether sodium; 96.77% w/w). For the PK and efficacy studies, the lyophilized powder was reconstituted with 19 mL sterile water for injection to yield RDV at 5 mg/mL in 15% SBECD in water pH 3.5 (tables S3 and S4). For the IV PK experiment in healthy AGM only, RDV (5 mg/mL) was formulated with 12% SBECD in sterile water (table S3). For the inhalation PK study and for the efficacy study, the RDV used for the inhalation and IV dosing groups was a 5 mg/mL cyclodextrin solution that is identical to the clinically-administered IV RDV. For bioanalysis, analyte standards GS-5734 (for RDV), GS-704277 (for the alanine metabolite), GS-441524 (for the nucleoside metabolite), and GS-443902 (for RDV-TP) were supplied by Gilead Sciences. ATP, ADP, AMP, and internal standards 5-(2-aminopropyl)indole (5-IT), chloro-ATP, and isotope-labeled nucleotides were purchased from Sigma-Aldrich. All reagents were at least of research grade.

### Animals

Wild-caught AGM (St. Kitts origin) were sourced through Worldwide Primates Inc. for all studies. Ages were unknown due to wild-caught sourcing, but were estimated to range from three to ten years based on dentition. All studies were conducted at Lovelace Biomedical Research Institute (LBRI). Model characterization and efficacy experiments involving AGM infected with SAR-CoV-2 were performed in an animal biosafety level 3 (ABSL-3) laboratory. All protocols were reviewed by an Institutional Animal Care and Use Committee (IACUC) at LBRI (IACUC #FY20-088). Research was conducted under an IACUC approved protocol in compliance with the Animal Welfare Act, PHS Policy, and other federal statutes and regulations relating to animals and experiments involving animals. The facilities where this research was conducted are accredited by the Association for Assessment and Accreditation of Laboratory Animal Care, International and strictly adhere to principles stated in the Guide for the Care and Use of Laboratory Animals, National Research Council, 2011 (National Academies Press, Washington, DC). All research methods and results are reported in compliance with the Animal Research: Reporting of In Vivo Experiments (ARRIVE) guidelines. AGM were housed in adjacent individual cages, in a climate-controlled room with a fixed light/dark cycle (12-hours light/12-hours dark). AGM were monitored at least twice daily throughout the study. Commercial monkey chow, treats, and fruit were provided twice daily by trained personnel. Water was available ad libitum. Animals were observed a minimum of twice daily for any clinical signs of test article toxicity or SARS-CoV-2 associated disease. A standard lexicon of pharmacologic and toxicologic observations was used for documentation of any abnormal clinical signs.

### Virus and cells

SARS-CoV-2 isolate USA-WA1/2020 was sourced from World Reference Center for Emerging Viruses and Arboviruses and passaged once in Vero E6 AGM kidney cells (BEI Resources) at University of Texas Medical Branch. Inoculation material was fifth passage from the original patient isolate. Virus was stored at -70°C or below until infections. For IN/IT instillations, virus was thawed, diluted with Dulbecco's Modified Eagle Medium (DMEM) to approximately 1×10^6^ TCID_50_/mL, and stored on ice until inoculations. Two aliquots of each inoculum were used for back-titration and confirmation of titer by TCID_50_ assay using Vero E6 cells.

The stock was sequenced at ATCC using the Illumina MiSeq next generation sequencing (NGS) platform. Relative to the reference strain (SARS-CoV-2/human/USA/USA-WA1/2020), there was one minority variant, a single nucleotide polymorphism (SNP) at nucleotide position 23525. This location is in the coding sequence for the SARS-CoV-2 Spike protein. Specifically, it is in the S1 portion of Spike, and is in the first position of the 655 codon, resulting in the non-conservative substitution, H655Y. The functional impacts of this mutation on SARS-CoV-2 entry, replication, or virulence in humans or animal models are currently unknown; however, Spike is functionally unrelated and genomically distant to nsp12, the target of RDV.

### Head dome RDV inhalation administration

For administration by inhalation, the solution formulation was aerosolized using a compressed air nebulizer (Pari LC Plus, PARI Respiratory Equipment, Inc.). Aerosolized RDV was delivered to alert, chair-restrained, pre-trained AGM directly to the top of a non-human primate head dome. The nebulizer flow rate was adjusted to achieve the target aerosol concentration. During the exposures, animals were allowed to breathe freely from the exposure atmosphere flowing through the head dome to buffer chamber. Animals from each dose group were exposed separately. Animals were not fasted prior to dosing.

### Aerosol characterization

Aerosol concentration in the test atmosphere was determined for samples collected on glass filters (47 mm diameter) directly from each head dome during the exposure at a nominal flow (0.3 to 0.5 L/min). For the single-dose PK study, the amount collected on the filter was quantified using both gravimetric and chemical analyses. For the efficacy study, RDV concentration was determined by gravimetric analysis only. For gravimetric analyses, filters were dried prior to weighing. For chemical analyses, the filters were placed in 7 mL glass vials for quantification of RDV by UPLC-UV. Particle size of aerosol was measured directly from the reference head dome during exposure using an Aerodynamic Particle Sizer (APS; TSI Inc.) or an InTox Mercer inertial impactor at a nominal flow rate at 2 L/min for a fixed duration. The aerosol mass median aerodynamic diameter and geometric standard deviation were determined at least once during each exposure. The presented dose (mg/kg) for each animal was calculated based on post-exposure filter analyses and estimated tidal volumes based on individual animal body weights obtained within 1 day of dosing. The pulmonary dose for each animal was estimated based on an assumed deposition fraction of 25%.

### Calculation of pulmonary deposited dose

Respiratory minute volume (RMV; liters per min) was calculated using the following allometric equation: RMV = 0.608 × BW^0.852^, where BW is the exposure day body weight in kilograms (*24*). The estimated dose was calculated using the following formula: Dose = (C × RMV × T × DF)/BW, where C (mg/mL) is the average aerosol concentration of RDV measured in the mixing chamber, T (min) is exposure time, and the deposition fraction (DF) is assumed to be 25% (*13*, *24*).

### Sample collection and processing

Blood samples for plasma were collected into K_2_EDTA tubes and placed on ice until processing. Blood samples for PBMC isolation were collected at room temperature into CPT vacutainer tubes containing sodium heparin for isolation. Samples in K_2_EDTA tubes were centrifuged at 1700 to 1800 ×*g* at 4°C for 10 min, and plasma was aliquoted. Samples in CPT tubes were processed according to the manufacturer’s instructions. Plasma and isolated PBMC samples were frozen immediately and stored at −70°C or below. Samples collected from SARS-CoV-2 infected animals were inactivated by organic solvent sterilization prior to bioanalysis.

For the single-dose PK study, at approximately 24 hours post-dose, animals were anesthetized for surgical collection of terminal respiratory tract tissues. Once at a deep plane of anesthesia, the distal aspect of the lower left lung lobe was excised within 1 min of the start of surgery, and was immediately placed into liquid nitrogen. Animals were then euthanized, and the remaining respiratory tract, liver, and kidney were obtained. For animals in the inhaled RDV group, nasal and nasopharyngeal mucosa were obtained from both the right and left nares using a curette and rinsed with phosphate buffered saline. All tissues and rinsed mucosa were immediately placed into liquid nitrogen and stored at −70°C or below until analysis. To prevent dephosphorylation of the RDV-triphosphate, all tissues were collected and frozen within 3 min of euthanasia. The thoracic trachea and left lung from all animals were fixed in 10% neutral buffered formalin for histopathologic evaluation.

Nasal and throat swabs were collected at 1, 2, 4, and 6 dpi using a cotton-tipped applicator presoaked in sterile saline. Swabs were placed in a tube containing 0.5 mL sterile saline, frozen immediately on dry ice, and stored at −70°C until analysis of viral titer. BALF was collected from left and right caudal lung lobes at 1, 2, and 4 dpi. Briefly, a pediatric bronchoscope (Olympus XP-40) was advanced into the caudal lung lobe, 10 mL of sterile saline was infused, and the maximum volume was aspirated. BALF was centrifuged at 1000 ×*g* for 10 min at 4°C. The resulting cell pellet and 1 mL aliquots of supernatant were frozen immediately and stored at −70°C until analysis or inactivation by organic solvent sterilization and subsequent bioanalysis.

For the SARS-CoV-2 model characterization and efficacy studies, animals were euthanized for tissue collection and necropsy at 6 dpi. Sections of the right lung were flash frozen in liquid nitrogen and stored at −70°C until analysis of viral titer. The thoracic trachea, left lung and tracheobronchial lymph nodes were fixed in 10% neutral buffered formalin (NBF) for histopathologic evaluation.

### Histopathology

The left lungs were instilled with 10% NBF until the pleura were tense ±20 s (to stimulate full physiological lung volume), and were then tied off at the trachea. Fixed tissues were trimmed, paraffin embedded, sectioned, and stained with hematoxylin and eosin. Microscopic examination was performed by a board-certified veterinary pathologist, and histologic lesions were scored based on the incidence, severity, and distribution of pathology. Histopathologic changes, as characterized by mixed cell inflammation and increased macrophages, were graded semiquantitatively by a single pathologist on a scale of 0 to 5 (1 = Minimal, 2 = Mild, 3 = Moderate, 4 = Marked, 5 = Severe).

### Organic solvent sterilization of samples from SARS-CoV-2 infected animals.

Inactivation of SARS-CoV-2-infected plasma and BALF supernatant samples was performed by treatment with 100% acetonitrile. Samples were incubated with acetonitrile at a 1:8 ratio for a minimum of 5 min on ice prior to removal from the ABSL-3 facility. Inactivation of SARS-CoV-2-infected PBMC and BALF cell pellet samples was performed by treatment with 70% methanol. PBMC or BALF cell pellets were mixed with methanol for a minimum of 5 min and were then centrifuged under vacuum to remove the liquid prior to removal from the ABSL-3 facility. Bioanalysis of the inactivated samples was performed using a liquid chromatography separation with tandem mass spectrometry detection (LC-MS/MS) as described above.

### Bioanalysis

Bioanalysis was performed using a liquid chromatography separation with tandem mass spectrometry detection (LC-MS/MS). For plasma samples, a 25 μL aliquot was treated with 200 μL of 100% acetonitrile with 20 nM 5-(2-aminopropyl)indole (5-IT; as the internal standard). The sample was filtered through an Agilent Captiva 96 well 0.2 μm filter plate. Filtered samples were dried down completely for approximately 30 min and reconstituted with 250 μL water; an aliquot of 10 μL was injected for LC-MS/MS analysis. Analytes were separated on a Phenomenex Synergi Hydro-RP 30A column (150 × 2.0 mM, 4.0 μm) at 25°C using a Waters Acquity Ultra Performance LC (Waters Corporation), a flow rate of 0.26 mL/min, and a gradient from mobile phase A (Water containing 0.2% formic acid) and 1% mobile phase B (acetonitrile/water, 95:5, containing 0.2% formic acid) over 4.5 min. MS/MS analyses used a Waters Xevo TQ-S triple quadrupole mass spectrometer (Waters Corporation) with a electrospray probe and analytes were detected in positive ion mode with a multiple reaction monitoring (MRM) method (*m/z* 603→200 for RDV, 443→202 for alanine metabolite, 292→163 for nucleoside metabolite and 393→261 for 5-IT). Plasma concentrations were determined using an 8-point calibration curve spanning a concentration range of over three orders of magnitude (3.3 to 830 nM for RDV, 11 to 11,300 nM for alanine metabolite and 6.9 to 3,430 nM for nucleoside metabolite). Quality control samples were run at the beginning and end of the run to ensure accuracy and precision within 20%.

PBMC samples were treated with 500 μL of dry ice-cold extraction buffer (70% methanol containing 0.1% KOH, 67 mM EDTA, as well as 250 nM chloro-adenosine triphosphate, chloro-ATP, as internal standard). The solution was vortexed for 5 min, then centrifuged at 20,000 ×g for 10 min. Supernatant was transferred to clean 1.5 mL Eppendorf vials and loaded onto a centrifuging evaporator. Once dry, samples were reconstituted with 80 μL of 1 mM ammonium dihydrogen phosphate buffer (pH 7), and transferred to high-performance liquid chromatography (HPLC) injection vials for analysis. An aliquot of 10 μL was injected into an LC-MS/MS system. An aliquot of 10 μL was injected for LC-MS/MS using an HTC Pal autosampler. Analytes were separated on a Phenomenex Luna C18 HST column (50 × 2.0 mM, 2.5 μm) using a ternary pump system (Shimadzu Scientific), a flow rate of 0.35 mL/min, and a gradient of Mobile phase A (Water containing 3 mM ammonium formate and 10 mM dimethylhexylamine, pH 5) and mobile phase B (acetonitrile/water, 50:50, containing 3 mM ammonium formate and 10 mM dimethylhexylamine, pH 5) over 6.0 min. MS/MS analyses used a triple-quadrupole mass spectrometer (Sciex QTrap 6500+; Sciex) in electrospray positive MRM method (*m/z* 532→202 for RDV-TP and 542→170 for chloro-ATP). PBMC concentrations of RDV-TP were determined using 6-point calibration curves spanning a concentration range of over three orders of magnitude (20 to 40,000 fmol/sample for the IV group and 40 to 40,000 fmol/sample for the inhalation groups). To calculate the intracellular concentration of metabolites, the total number of cells in each sample was quantified by a DNA-detection method (*25*). The intracellular micromolar concentrations were then derived using a cellular volume of 0.2 pL per cell.

Mucosa samples were treated with 500 μL of dry ice-cold extraction buffer (70% methanol containing 0.1% KOH, 67 mM EDTA, as well as 250 nM chloro-ATP as internal standard). The solution was vortexed for 15 min, then transferred to 1.5 mL Eppendorf vials and centrifuged at 20,000 ×*g* for 25 min. Supernatant was transferred to clean 1.5 mL Eppendorf vials and loaded onto a centrifuging evaporator. Once dry, samples were reconstituted with 80 μL of 1 mM ammonium dihydrogen phosphate buffer (pH 7) and transferred to HPLC injection vials for analysis. An aliquot of 10 μL was injected into an LC-MS/MS system and analyzed as described for PBMCs. Because mucosal samples were collected into sterile saline, concentrations of RDV and metabolites in these samples were expressed relative to endogenous ATP, and a qualitative analysis was performed by measuring the LC-MS/MS peak areas for RDV-TP and endogenous ATP.

For analysis of other tissues, each frozen sample was pulverized using a cell crusher on dry ice, transferred into a pre-weighed 15 mL conical tube, and weighed. A 4-fold volume of dry ice-cold extraction buffer (70% methanol containing 0.1% KOH, 67 mM EDTA, as well as 20 nM 5-IT and 250 nM chloro-ATP, as internal standards) was added. The mixture was then promptly homogenized using a tissue homogenizer with a disposable hard-tissue homogenizer probe (Omni Tip; Omni International). Standard curves (range 0.017 to 12.5 nmol/g tissue) were generated by spiking an appropriate amount of 100 μM of GS-441524, GS-704277, and GS-443902 solution, prepared in water, into blank tissue homogenates. A 200 μL aliquot of the homogenate was filtered using a 96-well filter plate (0.2 μm polypropylene, Agilent Captiva). The filtrate was evaporated to dryness and reconstituted with a 200 μL of 1 mM ammonium dihydrogen phosphate buffer (pH 7) prior to LC-MS/MS analysis. Blank (undosed) lung was used to build standard curves for quantification of the metabolites in respiratory tissues. Blank (undosed) liver was used for quantification of the metabolites in liver and kidney. To evaluate the potential dephosphorylation of the nucleotide metabolites in the tissue samples, endogenous AMP, ADP, and ATP were also quantified using isotope-labeled nucleotides as reference standards. The LC-MS/MS conditions for analyzing the metabolites in tissue samples was the same as used for PBMC analyses.

### Reverse transcription quantitative PCR (RT-qPCR) and infectious virus titers

Nasal and throat swabs, BALF samples, and trachea, bronchus, and lung tissue samples were assessed for SARS-CoV-2 RNA and infectious virus titers by RT-qPCR and TCID_50_ assay, respectively. For swabs and BALF, RNA was extracted from the liquid supernatants. Tissue samples (about 100 mg) were homogenized in lysis buffer using a TissueLyser (Qiagen), centrifuged at 10,000 ×*g* for 1 min; supernatants were collected for infectious virus titers and RNA extraction. RNA was isolated from all samples using the Direct-zol RNA purification kit (Zymo Research) according to the manufacturer’s instructions.

SARS-CoV-2 genome copies were quantified by RT-qPCR targeting the SARS-CoV-2 nucleocapsid phosphoprotein gene (N gene), and genome copies per mL or g equivalents were calculated from a standard curve. The SARS-CoV-2 N2 gene primers and probe sequences were as follows: forward primer 5′TTACAAACATTGGCCGCAAA3′; reverse primer 5′GCGCGACATTCCGAAGAA3′; probe: 6FAM-ACAATTTGCCCCCAGCGCTTCAG-BHQ-1. Amplification and detection were performed under the following thermal cycling conditions: 50°C for 5 min, 95°C for 20 s, 40 cycles of 95°C for 3 s, and 60°C for 30 s. All samples were analyzed in triplicate.

SARS-CoV-2 subgenomic copies were quantified by RT-qPCR targeting the SARS-CoV-2 E gene, and genome copies per milliliter or gram equivalents were calculated from a standard curve. The SARS-CoV-2 E gene primers and probe sequences were as follows: sgLead SARS-CoV-2 Forward: 5′ CGATCTCTTGTAGATCTGTTCTC 3′; E Sarbeco Reverse: 5′ ATATTGCAGCAGTACGCACACA 3′; E Sarbeco Probe: 6FAM-ACACTAGCCATCCTTACTGCGCTTCG-BHQ-1. Amplification and detection were performed under the following thermal cycling conditions: 50°C for 5 min, 95°C for 20 s, 40 cycles of 95°C for 3 s, and 60°C for 30 s. All samples were analyzed in triplicate.

Infectious virus titers were determined in a TCID_50_ assay using Vero E6 cells in a 96-well format. Vero E6 cells were plated on flat-bottom 96-well tissue culture plates to ≥ 90% confluency. Ten-fold serial dilutions of each sample were prepared in viral infection media (VIM) containing DMEM + 2% fetal bovine serum + 1% Pen/Strep. 100 μL of each sample dilution was plated per well. Each sample was assayed in replicates of five, and plates were incubated at 37°C for approximately 72 hours or until cytopathic effect (CPE) was discernable. Stock virus of known concentration and blank VIM served as positive and negative controls, respectively. At assay completion, cells were fixed with 50 μL of 4% formalin per well for a minimum of 2 hours at 2 to 8°C. Fixed cells were stained with 10 to 20 μL Crystal Violet per well for a minimum of one hour at room temperature. CPE was visually assessed for each well, and the TCID_50_ titer was calculated according to the Reed-Muench method.

### Statistical analyses

All raw, individual-level data are presented in data file S1. Statistical analyses were performed using GraphPad Prism software. For all endpoints, inhaled and IV treatment routes were analyzed independently. For longitudinal analyses, RDV treatment was compared with its respective vehicle treatment by repeated-measures two-way ANOVA with Bonferroni post-hoc correction for multiple comparisons. For single timepoint analyses, RDV treatment was compared with its respective vehicle treatment by two-tailed *t* tests. Samples analyzed by RT-qPCR, and TCID_50_ that were below the assay lower limit of quantification were assigned a value equal to the lower limit of quantification. For all analyses, a corrected *p* value <0.05 was considered statistically significant. Pharmacokinetic analyses, including determination of the C_max_ and AUC of RDV and metabolites, were performed using Phoenix WinNonlin software.

The sample size calculation for the efficacy study was based on the variability observed in key viral quantification endpoints from the pilot study. Based on a desired treatment effect size of 1.5, a study with 80% power required 6 animals per group to detect treatment effects using an alpha cut-off of 5%. In the IV treatment group for the PK study, results from two samples collected at 0.25 hours post-dose were excluded due to the samples being collected from the infusion site, resulting in artificially high blood concentrations of RDV and metabolites. No other data points were excluded from analyses.

## Supplementary Materials

### Other Supplementary Material for this manuscript includes the following:

Data file S1

MDAR Reproducibility Checklist

## References

[1] E. de Wit, N. van Doremalen, D. Falzarano, V. J. Munster, SARS and MERS: Recent insights into emerging coronaviruses. Nat. Rev. Microbiol. 14, 523–534 (2016). 10.1038/nrmicro.2016.81 27344959 PMC7097822

[2] T. G. Ksiazek, D. Erdman, C. S. Goldsmith, S. R. Zaki, T. Peret, S. Emery, S. Tong, C. Urbani, J. A. Comer, W. Lim, P. E. Rollin, S. F. Dowell, A.-E. Ling, C. D. Humphrey, W.-J. Shieh, J. Guarner, C. D. Paddock, P. Rota, B. Fields, J. DeRisi, J.-Y. Yang, N. Cox, J. M. Hughes, J. W. LeDuc, W. J. Bellini, L. J. Anderson; SARS Working Group, A novel coronavirus associated with severe acute respiratory syndrome. N. Engl. J. Med. 348, 1953–1966 (2003). 10.1056/NEJMoa030781 12690092

[3] A. Assiri, J. A. Al-Tawfiq, A. A. Al-Rabeeah, F. A. Al-Rabiah, S. Al-Hajjar, A. Al-Barrak, H. Flemban, W. N. Al-Nassir, H. H. Balkhy, R. F. Al-Hakeem, H. Q. Makhdoom, A. I. Zumla, Z. A. Memish, Epidemiological, demographic, and clinical characteristics of 47 cases of Middle East respiratory syndrome coronavirus disease from Saudi Arabia: A descriptive study. Lancet Infect. Dis. 13, 752–761 (2013). 10.1016/S1473-3099(13)70204-4 23891402 PMC7185445

[4] W. S. Choi, C. I. Kang, Y. Kim, J. P. Choi, J. S. Joh, H. S. Shin, G. Kim, K. R. Peck, D. R. Chung, H. O. Kim, S. H. Song, Y. R. Kim, K. M. Sohn, Y. Jung, J. H. Bang, N. J. Kim, K. S. Lee, H. W. Jeong, J.-Y. Rhee, E. S. Kim, H. Woo, W. S. Oh, K. Huh, Y. H. Lee, J. Y. Song, J. Lee, C.-S. Lee, B.-N. Kim, Y. H. Choi, S. J. Jeong, J.-S. Lee, J. H. Yoon, Y. M. Wi, M. K. Joung, S. Y. Park, S. H. Lee, S.-I. Jung, S.-W. Kim, J. H. Lee, H. Lee, H. K. Ki, Y.-S. Kim; Korean Society of Infectious Diseases, Clinical Presentation and Outcomes of Middle East Respiratory Syndrome in the Republic of Korea. Infect. Chemother. 48, 118–126 (2016). 10.3947/ic.2016.48.2.118 27433382 PMC4945721

[5] Who Mers-Cov Research Group, State of Knowledge and Data Gaps of Middle East Respiratory Syndrome Coronavirus (MERS-CoV) in Humans. PLOS Curr. 5, ••• (2013). 24270606 10.1371/currents.outbreaks.0bf719e352e7478f8ad85fa30127ddb8 PMC3828229

[6] N. Zhu, D. Zhang, W. Wang, X. Li, B. Yang, J. Song, X. Zhao, B. Huang, W. Shi, R. Lu, P. Niu, F. Zhan, X. Ma, D. Wang, W. Xu, G. Wu, G. F. Gao, W. Tan; China Novel Coronavirus Investigating and Research Team, A Novel Coronavirus from Patients with Pneumonia in China, 2019. N. Engl. J. Med. 382, 727–733 (2020). 10.1056/NEJMoa2001017 31978945 PMC7092803

[7] Z. Wu, J. M. McGoogan, Characteristics of and Important Lessons From the Coronavirus Disease 2019 (COVID-19) Outbreak in China: Summary of a Report of 72 314 Cases From the Chinese Center for Disease Control and Prevention. JAMA 323, 1239–1242 (2020). 10.1001/jama.2020.2648 32091533

[8] R. L. Mackman, H. C. Hui, M. Perron, E. Murakami, C. Palmiotti, G. Lee, K. Stray, L. Zhang, B. Goyal, K. Chun, D. Byun, D. Siegel, S. Simonovich, V. Du Pont, J. Pitts, D. Babusis, A. Vijjapurapu, X. Lu, C. Kim, X. Zhao, J. Chan, B. Ma, D. Lye, A. Vandersteen, S. Wortman, K. T. Barrett, M. Toteva, R. Jordan, R. Subramanian, J. P. Bilello, T. Cihlar, Prodrugs of a 1′-CN-4-Aza-7,9-dideazaadenosine *C*-Nucleoside Leading to the Discovery of Remdesivir (GS-5734) as a Potent Inhibitor of Respiratory Syncytial Virus with Efficacy in the African Green Monkey Model of RSV. J. Med. Chem. 64, 5001–5017 (2021). 10.1021/acs.jmedchem.1c00071 33835812

[9] T. K. Warren, R. Jordan, M. K. Lo, A. S. Ray, R. L. Mackman, V. Soloveva, D. Siegel, M. Perron, R. Bannister, H. C. Hui, N. Larson, R. Strickley, J. Wells, K. S. Stuthman, S. A. Van Tongeren, N. L. Garza, G. Donnelly, A. C. Shurtleff, C. J. Retterer, D. Gharaibeh, R. Zamani, T. Kenny, B. P. Eaton, E. Grimes, L. S. Welch, L. Gomba, C. L. Wilhelmsen, D. K. Nichols, J. E. Nuss, E. R. Nagle, J. R. Kugelman, G. Palacios, E. Doerffler, S. Neville, E. Carra, M. O. Clarke, L. Zhang, W. Lew, B. Ross, Q. Wang, K. Chun, L. Wolfe, D. Babusis, Y. Park, K. M. Stray, I. Trancheva, J. Y. Feng, O. Barauskas, Y. Xu, P. Wong, M. R. Braun, M. Flint, L. K. McMullan, S.-S. Chen, R. Fearns, S. Swaminathan, D. L. Mayers, C. F. Spiropoulou, W. A. Lee, S. T. Nichol, T. Cihlar, S. Bavari, Therapeutic efficacy of the small molecule GS-5734 against Ebola virus in rhesus monkeys. Nature 531, 381–385 (2016). 10.1038/nature17180 26934220 PMC5551389

[10] R. Li, A. Liclican, Y. Xu, J. Pitts, C. Niu, J. Zhang, C. Kim, X. Zhao, D. Soohoo, D. Babusis, Q. Yue, B. Ma, B. P. Murray, R. Subramanian, X. Xie, J. Zou, J. P. Bilello, L. Li, B. E. Schultz, R. Sakowicz, B. J. Smith, P. Y. Shi, E. Murakami, J. Y. Feng, Key Metabolic Enzymes Involved in Remdesivir Activation in Human Lung Cells. Antimicrob. Agents Chemother. 65, e0060221 (2021). 10.1128/AAC.00602-21 34125594 PMC8370248

[11] US Food and Drug Administration, News Release: FDA Approves First Treatment for COVID-19. 22 October 2020. Available at: https://www.fda.gov/news-events/press-announcements/fda-approves-first-treatment-covid-19.

[12] R. Wölfel, V. M. Corman, W. Guggemos, M. Seilmaier, S. Zange, M. A. Müller, D. Niemeyer, T. C. Jones, P. Vollmar, C. Rothe, M. Hoelscher, T. Bleicker, S. Brünink, J. Schneider, R. Ehmann, K. Zwirglmaier, C. Drosten, C. Wendtner, Virological assessment of hospitalized patients with COVID-2019. Nature 581, 465–469 (2020). 10.1038/s41586-020-2196-x 32235945

[13] J. S. Tepper, P. J. Kuehl, S. Cracknell, K. J. Nikula, L. Pei, J. D. Blanchard, Symposium Summary: “Breathe In, Breathe Out, Its Easy: What You Need to Know About Developing Inhaled Drugs”. Int. J. Toxicol. 35, 376–392 (2016). 10.1177/1091581815624080 26857693

[14] R. Humeniuk, A. Mathias, H. Cao, A. Osinusi, G. Shen, E. Chng, J. Ling, A. Vu, P. German, Safety, Tolerability, and Pharmacokinetics of Remdesivir, An Antiviral for Treatment of COVID-19, in Healthy Subjects. Clin. Transl. Sci. 13, 896–906 (2020). 10.1111/cts.12840 32589775 PMC7361781

[15] B. N. Williamson, F. Feldmann, B. Schwarz, K. Meade-White, D. P. Porter, J. Schulz, N. van Doremalen, I. Leighton, C. K. Yinda, L. Pérez-Pérez, A. Okumura, J. Lovaglio, P. W. Hanley, G. Saturday, C. M. Bosio, S. Anzick, K. Barbian, T. Cihlar, C. Martens, D. P. Scott, V. J. Munster, E. de Wit, Clinical benefit of remdesivir in rhesus macaques infected with SARS-CoV-2. Nature 585, 273–276 (2020). 10.1038/s41586-020-2423-5 32516797 PMC7486271

[16] C. Woolsey, V. Borisevich, A. N. Prasad, K. N. Agans, D. J. Deer, N. S. Dobias, J. C. Heymann, S. L. Foster, C. B. Levine, L. Medina, K. Melody, J. B. Geisbert, K. A. Fenton, T. W. Geisbert, R. W. Cross, Establishment of an African green monkey model for COVID-19 and protection against re-infection. Nat. Immunol. 22, 86–98 (2021). 10.1038/s41590-020-00835-8 33235385 PMC7790436

[17] J. H. Beigel, K. M. Tomashek, L. E. Dodd, A. K. Mehta, B. S. Zingman, A. C. Kalil, E. Hohmann, H. Y. Chu, A. Luetkemeyer, S. Kline, D. Lopez de Castilla, R. W. Finberg, K. Dierberg, V. Tapson, L. Hsieh, T. F. Patterson, R. Paredes, D. A. Sweeney, W. R. Short, G. Touloumi, D. C. Lye, N. Ohmagari, M.-D. Oh, G. M. Ruiz-Palacios, T. Benfield, G. Fätkenheuer, M. G. Kortepeter, R. L. Atmar, C. B. Creech, J. Lundgren, A. G. Babiker, S. Pett, J. D. Neaton, T. H. Burgess, T. Bonnett, M. Green, M. Makowski, A. Osinusi, S. Nayak, H. C. Lane; ACTT-1 Study Group Members, Remdesivir for the Treatment of Covid-19 - Final Report. N. Engl. J. Med. 383, 1813–1826 (2020). 10.1056/NEJMoa2007764 32445440 PMC7262788

[18] J. Hill, R. Paredes Deiros, C. Carlos Vaca, J. Mera, B. Webb, G. Perez, P. Ryan-Murua, J. Gerstoft, M. Brown, G. Oguchi, J. Schiffer, S. Brown, M. Katz, A. Ginde, G. Camus, D. Porter, R. Hyland, S. Chen, K. Juneja, A. Osinusi, F. Duff, R. Gottlieb, Remdesivir for the treatment of high-risk non-hospitalized individuals with COVID-19: a randomized, double-blind, placebo-controlled trial. Abstract LB1, IDWeek 2021 Virtual Conference, 29 Sep – Oct 3 (2021).

[19] R. V. Blair, M. Vaccari, L. A. Doyle-Meyers, C. J. Roy, K. Russell-Lodrigue, M. Fahlberg, C. J. Monjure, B. Beddingfield, K. S. Plante, J. A. Plante, S. C. Weaver, X. Qin, C. C. Midkiff, G. Lehmicke, N. Golden, B. Threeton, T. Penney, C. Allers, M. B. Barnes, M. Pattison, P. K. Datta, N. J. Maness, A. Birnbaum, T. Fischer, R. P. Bohm, J. Rappaport, Acute Respiratory Distress in Aged, SARS-CoV-2-Infected African Green Monkeys but Not Rhesus Macaques. Am. J. Pathol. 191, 274–282 (2021). 10.1016/j.ajpath.2020.10.016 33171111 PMC7648506

[20] V. J. Munster, F. Feldmann, B. N. Williamson, N. van Doremalen, L. Pérez-Pérez, J. Schulz, K. Meade-White, A. Okumura, J. Callison, B. Brumbaugh, V. A. Avanzato, R. Rosenke, P. W. Hanley, G. Saturday, D. Scott, E. R. Fischer, E. de Wit, Respiratory disease in rhesus macaques inoculated with SARS-CoV-2. Nature 585, 268–272 (2020). 10.1038/s41586-020-2324-7 32396922 PMC7486227

[21] E. Speranza, B. N. Williamson, F. Feldmann, G. L. Sturdevant, L. Pérez-Pérez, K. Meade-White, B. J. Smith, J. Lovaglio, C. Martens, V. J. Munster, A. Okumura, C. Shaia, H. Feldmann, S. M. Best, E. de Wit, Single-cell RNA sequencing reveals SARS-CoV-2 infection dynamics in lungs of African green monkeys. Sci. Transl. Med. 13, eabe8146 (2021). 10.1126/scitranslmed.abe8146 33431511 PMC7875333

[22] A. L. Hartman, S. Nambulli, C. M. McMillen, A. G. White, N. L. Tilston-Lunel, J. R. Albe, E. Cottle, M. D. Dunn, L. J. Frye, T. H. Gilliland, E. L. Olsen, K. J. O’Malley, M. M. Schwarz, J. A. Tomko, R. C. Walker, M. Xia, M. S. Hartman, E. Klein, C. A. Scanga, J. L. Flynn, W. B. Klimstra, A. K. McElroy, D. S. Reed, W. P. Duprex, SARS-CoV-2 infection of African green monkeys results in mild respiratory disease discernible by PET/CT imaging and shedding of infectious virus from both respiratory and gastrointestinal tracts. PLOS Pathog. 16, e1008903 (2020). 10.1371/journal.ppat.1008903 32946524 PMC7535860

[23] J. D. Pitts, X. Lu, V. Du Pont, N. C. Riola, J. Li, S. Manhas, R. Martin, J. Perry, T. Cihlar, D. P. Porter, J. P. Bilello. Remdesivir Retains Potent Antiviral Activity Against the SARS-CoV-2 Delta Variant and Other Variants of Concern. Poster 135. ISIRV-WHO Virtual Conference, 19–21 October (2021).

[24] D. J. Alexander, C. J. Collins, D. W. Coombs, I. S. Gilkison, C. J. Hardy, G. Healey, G. Karantabias, N. Johnson, A. Karlsson, J. D. Kilgour, P. McDonald, Association of Inhalation Toxicologists (AIT) working party recommendation for standard delivered dose calculation and expression in non-clinical aerosol inhalation toxicology studies with pharmaceuticals. Inhal. Toxicol. 20, 1179–1189 (2008). 10.1080/08958370802207318 18802802

[25] H. Benech, F. Théodoro, A. Herbet, N. Page, D. Schlemmer, A. Pruvost, J. Grassi, J.-R. Deverre, Peripheral blood mononuclear cell counting using a DNA-detection-based method. Anal. Biochem. 330, 172–174 (2004). 10.1016/j.ab.2004.03.015 15183777

