# Supplementary material for: Inhaled remdesivir reduces viral burden in a nonhuman primate model of SARS-CoV-2 infection
Source: Sci Transl Med. 2021 Dec 30:eabl828. doi: 10.1126/scitranslmed.abl8282 (PMC8961622; doi:10.1126/scitranslmed.abl8282)
Supplement: Supplementary file 1 — Figs. S1 to S7 Tables S1 to S4 [file scitranslmed.abl8282_sm.pdf]

Science Translational Medicine
AAAS

Supplementary Materials for

Inhaled remdesivir reduces viral burden in a nonhuman primate model of SARS-CoV-2 infection

Meghan S. Vermillion *et al.*

Corresponding author: Danielle P. Porter, Danielle.Porter@gilead.com

DOI: 10.1126/scitranslmed.abl8282

The PDF file includes:

Figs. S1 to S7
Tables S1 to S4

Other Supplementary Material for this manuscript includes the following:

Data file S1
MDAR Reproducibility Checklist

Inhaled remdesivir reduces viral burden in a nonhuman primate model of SARS-CoV-2 infection

Supplementary Materials

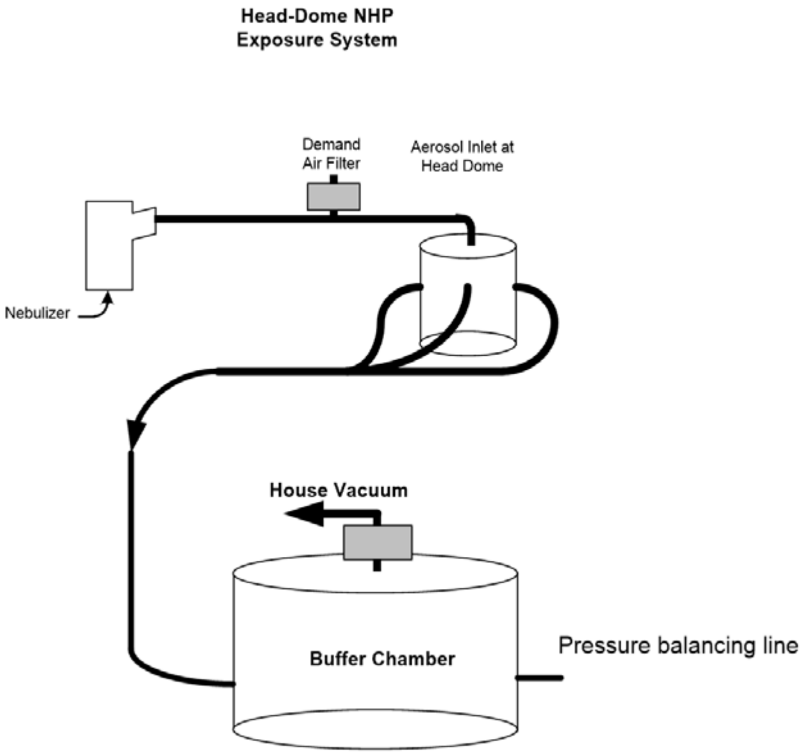

Fig. S1. Non-human primate (NHP) head dome exposure system schematic. Remdesivir (RDV) or vehicle were aerosolized using a compressed air nebulizer attached to the top of the head dome. Each head dome had multiple exhaust ports along the circumference of the head dome, which combined to form a manifold connected to a buffer chamber that connected to vacuum exhaust. The system includes a demand air filter and pressure balancing line in order to manage differences in pressure relative to animal respiration rate and timing and the exposure flows. Aerosol sampling (concentration and particle size) were collected from the head dome by a sampling port (not depicted).

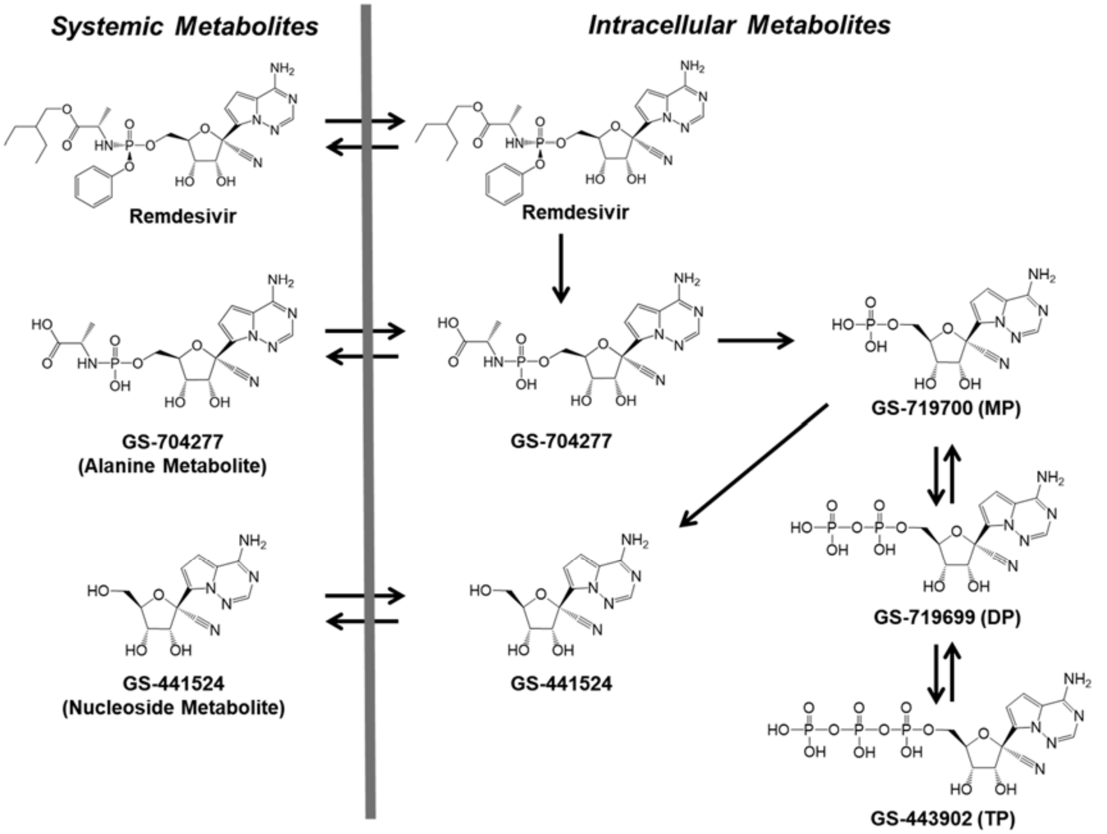

Fig. S2. Remdesivir (RDV) and its systemic and intracellular metabolites. RDV undergoes metabolic activation by the cellular enzymes to its pharmacologically active triphosphate (TP) form. MP, monophosphate; DP, diphosphate.

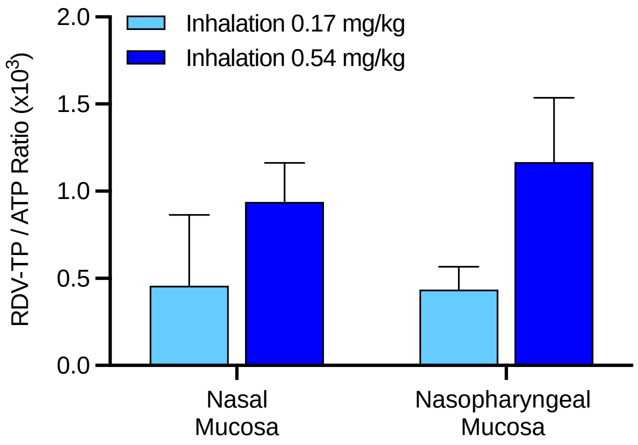

Fig. S3. Remdesivir triphosphate (RDV-TP) in the upper respiratory tract following RDV inhalation. Mean peak area ratios of RDV-TP:adenosine triphosphate(ATP) were measured from nasal and nasopharyngeal mucosa following inhaled delivery of 0.17 and 0.54 mg/kg deposited doses of RDV to assess the distribution and activation of RDV in the upper respiratory tract. Data are presented as mean + SD.

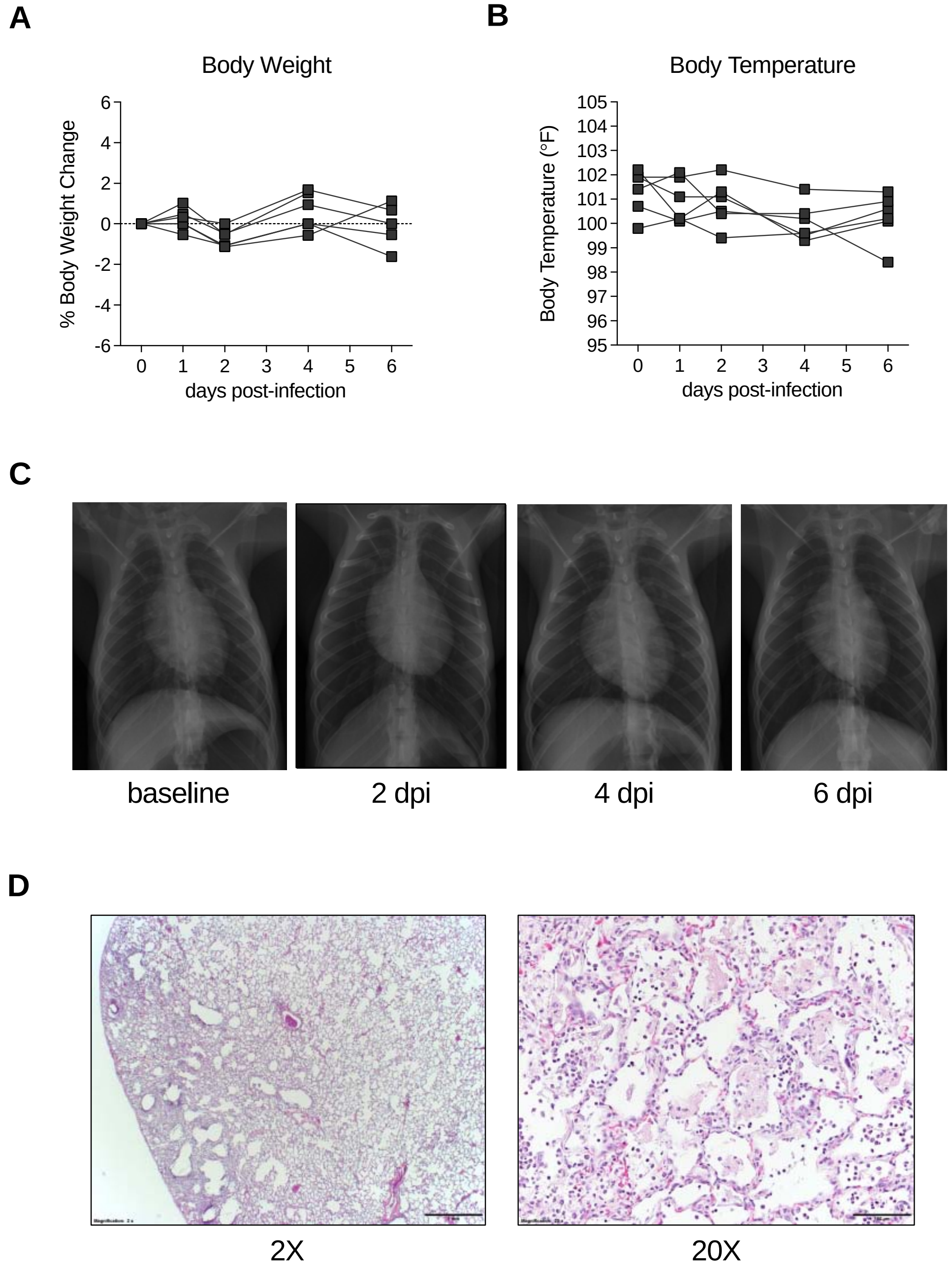

Fig. S4. Clinical and histopathologic changes following SARS-CoV-2 infection of African green monkeys (AGM). (A to C) Body weight change (A), body temperatures (B) and thoracic radiographs were collected at baseline and 2, 4 and 6 days post-infection (dpi) (C) following SARS-CoV-2 infection of AGM. (**D**) Representative photomicrographs show pulmonary inflammation and lung histopathology present 6 dpi. Scale bars represent 1 mm for 2× magnification and 100 µm for 20× magnification. Body weight data are reported as the percentage change compared with baseline values.

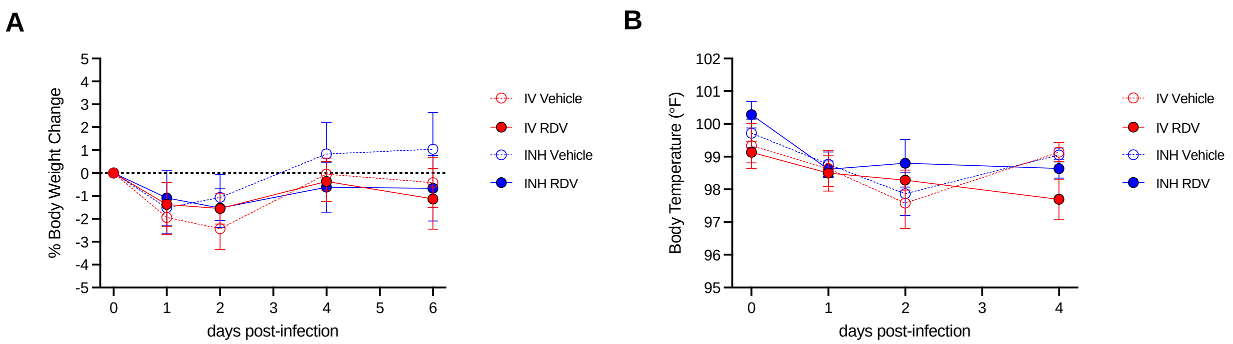

Fig. S5. Body weights and temperatures following SARS-CoV-2 infection and RDV or vehicle treatment. (**A and B**) Body weight change (A) and body temperatures (B) were recorded following SARS-CoV-2 infection and either RDV or vehicle treatment by either intravenous (IV) or inhalation (INH) administration. Group means ± SEM are presented. Body weight data are reported as the percentage change compared with baseline values.

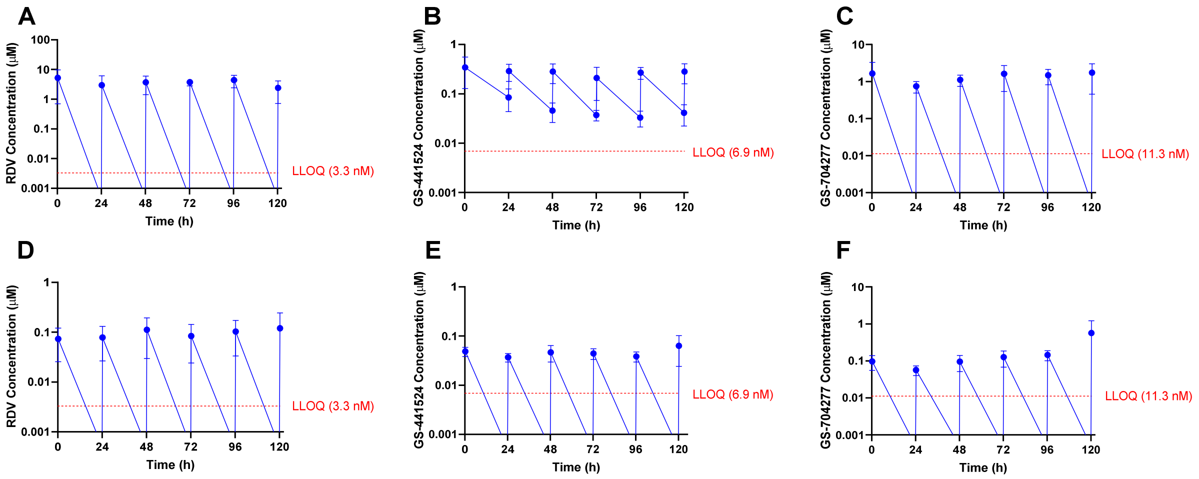

Fig. S6. Plasma pharmacokinetics (PK) of RDV, GS-441524 nucleoside metabolite, and GS-704277 alanine metabolite after SARS-CoV-2 infection and RDV treatment. (**A**) RDV, (**B**) GS-441524 nucleoside metabolite, and (**C**) GS-704277 alanine metabolite concentrations were measured following IV administration; (**D**) RDV, (**E**) GS-441524 nucleoside metabolite, and (**F**) GS-704277 alanine metabolite concentrations were measured following inhalation administration. LLOQ, lower limit of quantification.

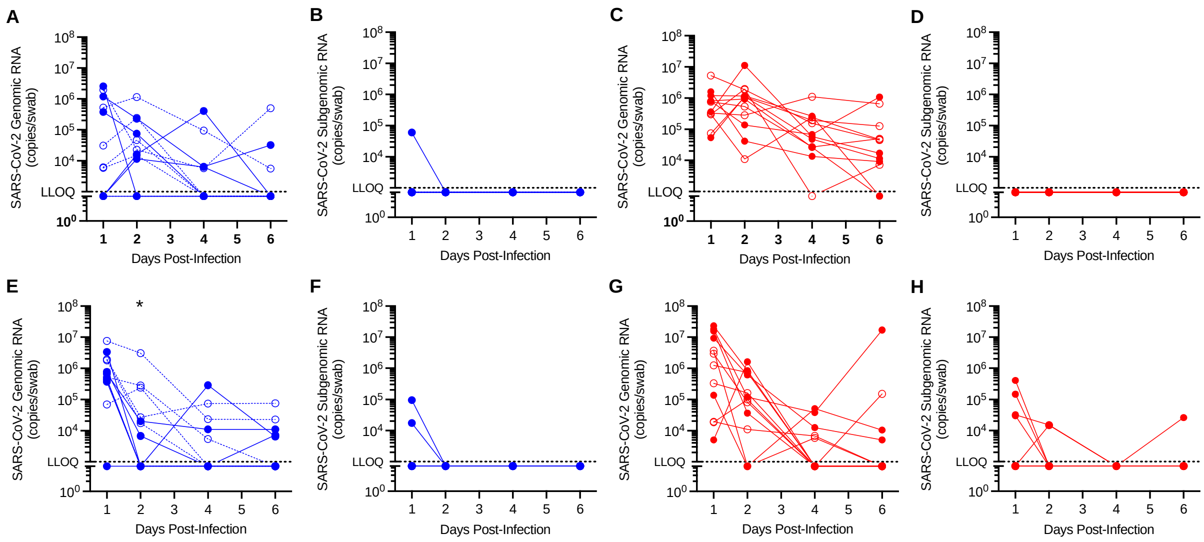

Fig. S7. Upper respiratory tract virus and infectious virus loads were measured following SARS-CoV-2 infection and RDV or vehicle treatment. AGM were inoculated with SARS-CoV-2 and treated with inhaled (blue symbols) or IV (red symbols) RDV (closed symbols) or vehicle (open symbols) beginning at 8 hours post-infection (n=6 per group). (**A to D**) Nasal and (**E to H**) throat SARS-CoV-2 genomic (**A, C, E, G**) and subgenomic (**B, D, F, H**) RNA copies were quantified by quantitative PCR. Samples that were below the lower limit of quantification (LLOQ, dotted lines) for the assay were assigned the LLOQ for analyses. *P<0.05; data were analyzed by a repeated measures two-way ANOVA with a Bonferroni post-hoc correction.

Table S1. Tissue and peripheral blood mononuclear cell (PBMC) concentrations of RDV-TP at 24 hours post dose. Results represent mean ± SD for IV group (n=3) and inhalation groups (n=4).

**RDV Route, Dose**	**RDV-TP Concentration (nmol/g tissue or μM in PBMC)**							
	**Upper trachea**	**Lower trachea**	**Upper Bronchi**	**Lower Bronchi**	**Lung**	**Liver***	**Kidney***	**PBMC**
IV, 10 mg/kg	0.54 ± 0.15	0.54 ± 0.15	0.54 ± 0.15	0.54 ± 0.15	1.03 ± 0.19	17.3 ± 2.72	39.8 ± 2.46	7.54
Inhalation, 0.17 mg/kg	0.069 ± 0.029	0.091 ± 0.058	0.762 ± 0.360	0.562 ± 0.105	0.518 ± 0.225	0.446 ± 0.299	0.445 ± 0.109	0.047 ± 0.023
Inhalation, 0.54 mg/kg	0.208 ± 0.167	0.266 ± 0.251	1.99 ± 1.71	1.61 ± 1.20	1.58 ± 1.64	0.695 ± 0.196	1.23 ± 0.20	0.127 ± 0.040

* Total nucleotide concentration (sum of nucleoside, MP, DP, and TP)

Table S2. Left lung lobe pathology results following SARS-CoV-2 infection of AGM. Left lung pathology results for n=2 males and n=4 females per group. The highest degree of severity recorded in cranial and caudal lung lobe is listed.

	**Males**				**Females**			
	**Vehicle**		**RDV**		**Vehicle**		**RDV**	
	**IV**	**Inhaled**	**IV**	**Inhaled**	**IV**	**Inhaled**	**IV**	**Inhaled**
Inflammation, mixed cell (focal/multifocal)								
Incidence	2	2	2	1	4	4	2	2
Minimal – 1	1	1	2	1	3	3	2	1
Mild – 2	1	1	–	–	1	1	–	1
Increase, macrophages								
Incidence	1	1	0	2	0	1	0	1
Minimal – 1	1	1	–	2	–	1	–	1

Table S3. Study design of single-dose RDV pharmacokinetics. NA, not applicable.

Route of Administration	Formulation	Dose	Head Dome Exposure Time	No. of Animals per Sex
IV,<br>30 min infusion	RDV lyophilized powder reconstituted to obtain 5 mg/mL RDV, 12% sulfobutylether-β-cyclodextrin in water (pH 3.5)	10 mg/kg	NA	3 males
Head dome inhalation	RDV lyophilized powder reconstituted to obtain 5 mg/mL RDV, 15% sulfobutylether-β-cyclodextrin in water (pH 3.6)	0.17 mg/kg mean deposited	30 minutes	3 males,<br>1 female
		0.54 mg/kg mean deposited	90 minutes	3 males,<br>1 female

Table S4. Study design of RDV efficacy in SARS-CoV-2 AGM model. The study was conducted in 3 cohorts of n=8 animals per cohort (2 per group), staggered by 1 day each. NA, not applicable.

Route of Administration	Formulation	Dose	Head Dome Exposure Time	No. of Animals/Sex
IV, 30 min infusion	15% sulfobutylether-β-cyclodextrin in water (pH 3.5)	0	NA	2 males, 4 females
IV, 30 min infusion	RDV lyophilized powder reconstituted to obtain 5 mg/mL RDV, 15% sulfobutylether-β-cyclodextrin in water (pH 3.5)	10 mg/kg beginning ~8 hours post-inoculation and 5 mg/kg once daily thereafter for 6 days total	NA	2 males, 4 females
Head dome inhalation	15% sulfobutylether-β-cyclodextrin in water (pH 3.5)	0	60 minutes	2 males, 4 females
Head dome inhalation	RDV lyophilized powder reconstituted to obtain 5 mg/mL RDV, 15% sulfobutylether-β-cyclodextrin in water (pH 3.5)	0.35 mg/kg average daily deposited dose beginning ~8 hours post-inoculation and once daily thereafter for 6 days total	60 minutes	2 males, 4 females
